# Retro-Pharyngeal Abscess—Report of Two Cases

**Published:** 1887-09

**Authors:** J. M. Crawford

**Affiliations:** Assistant to Chair of Eye, Ear and Throat Diseases in the Atlanta Medical College, Atlanta, Georgia


### Clinic Reports.

### RETRO-PHARYNGEAL ABSCESS—REPORT OF TWO CASES.

BY J. M. CRAWFORD, M. D., ASSISTANT TO CHAIR OF EYE, EAR AND THROAT DISEASES IN THE ATLANTA MEDICAL COLLEGE, ATLANTA, GEORGIA.

Retro-pharyngeal abscess is of uncommon occurrence, and on account of its rarity, these two cases are given to the public:

*Case 1.*—A. S., farmer, 50 years old, presented himself October 15, 1886. He had been a great sufferer from rheumatism for several months past, and three weeks before applying for treatment for his present trouble, his throat had pained him very much.

He had been treated by another physician, but had received no benefit. When I saw him, his appearance was that of a man who had been suffering from typhoid fever. He was very much emaciated and walked with difficulty. He complained of great pain in the throat. He spoke with difficulty and only in a whisper.

On examination I found the posterior portion of pharynx bulged forward, putting the mucous membrane on the stretch. The throat was almost entirely closed. Suspecting an abscess, I introduced an exploring needle and found pus. With an ordinary bistoury I punctured the upper portion of the abscess, making a free opening, frcm which quite a quantity of pus escaped. I cut downwards into it, bringing my knife out at the lowest part in order to prevent the flow of pus into the larynx.

If the cut had been made from below upwards, there would have been greater danger of the pus going into the larynx. As soon as the cut was made, I had him to lower his head in order to allow the pus to flow from his mouth, which it did very freely. The patient was directed to take twenty drops of muriated tincture of iron and a toddy three times daily. He presented himself again at the end of a week, when I found that the opening I had made had closed and there was evidence of a re-accumulation of pus in the same cavity; again the bistoury was resorted to and a freer opening was made than before, when there was again a free discharge of pus. From this time on there was no tendency of the wound to heal, and the discharge was kept up for several weeks, the patient being kept on the same treatment as already mentioned. At this writing the patient is entirely well.

*Case* 2.—D. W., 20 years old, very much broken in health, applied to me January 20, 1887, for a throat trouble that he had had for quite a while. It was impossible to get any definite history as to the exact date of the throat trouble. He gave a history of syphilis of several months' standing.

Examination revealed no syphilitic lesions, except the throat trouble, and that did not have the appearance of an ordinary syphilitic throat. The pharynx was very much swollen, bulging forward so much as to render deglutition difficult. He complained of great pain; said that the suffering was so great that he could not sleep at night. He had for several days been unable to take any food, except a liquid diet, and this always gave him great pain in swallowing. When these cases have once been seen, there is very little danger of mistaking them for anything else. I suspected an abscess as soon as I examined well the parts, and introduced an exploring needle, which brought away pus. A free incision was then made into the sac, and a considerable quantity of offensive pus was discharged.

The patient was put on anti-syphilitic treatment, and soon made a good recovery.

